# Metal-Organic Frameworks for Diabetic Wound Healing

**DOI:** 10.7759/cureus.39557

**Published:** 2023-05-27

**Authors:** Mohammad A Alghamdi

**Affiliations:** 1 Internal Medicine, Albaha University, Albaha, SAU

**Keywords:** zinc, copper, cobalt, wound healing, diabetes, mofs

## Abstract

Wound healing is one of the most important issues in clinical and scientific research. The healing process is complex and requires many agents to overcome in a short duration. A recent class of porous materials called metal-organic frameworks (MOFs) has great potential towards improving wound healing. This is attributed to their well-designed structures with large surface areas amenable to cargo loading and adjustable pore size ready for biological implementations. MOFs are assembled by several metal centers and organic linkers. In particular, metal ions can be released from MOFs when they are degraded in the biological environment. This endows MOF-based systems with dual functions to typically shorten the healing duration. This work focuses on using MOFs with different metal centers such as copper (Cu), zinc (Zn), cobalt (Co), magnesium (Mg), and zirconium (Zr) for healing diabetic wounds as one of the most required clinical issues to be resolved. By figuring out the presented examples of this work, several potential research ideas can emerge to explore new porous materials or even new MOFs for more control over the healing process.

## Introduction and background

Repairing injured tissue or skin regeneration and tissue restoration of normal structures requires a complex, demanding, and highly controlled biological process governed by a number of different variables [1, 2]. There are two primary categories of wounds - acute and chronic - distinguished by healing duration and infection severity. Full-thickness wounds include destruction to the subcutaneous tissue, whereas acute wounds affect the epidermis and superficial dermis [3]. Acute wounds are ones that begin suddenly and proceed through the stages of healing as predicted. For example, incisions made during surgery, abrasions, cuts, and lacerations, all of which have the potential to get infected but are also capable of completing the full healing process within the allotted time period [4].

Wound healing is a multi-step process that may become dysfunctional if interrupted at any point. Chronic wounds are those that have been inflamed for an extended period of time (months or years), prolonging the healing process and raising the risk of complications such as infection and elevated protease activity [5]. Wounds that are considered "acute" can usually heal in a matter of weeks, but chronic wounds take longer than three months to heal [6]. Chronic wounds result from damage to the skin's three layers (the epidermis, dermis, as well as subcutaneous adipose tissue). Most chronic wounds are secondary consequences of other diseases, such as diabetic foot ulcers (DFUs), pressure sores from spinal cord injuries, or even difficulties in wound care due to chronic neurological diseases like Parkinson's disease.

High morbidity and medical costs are connected with diabetic wounds, making them a severe serious consequence of diabetes mellitus [7, 8]. Diabetic wound patients are consistently unable to heal using typical conventional treatments due to factors like unsatisfactory angiogenesis, chronic inflammation, abnormal collagen metabolism, etc. Accordingly, the search for effective therapy towards diabetic wounds remains a significant dilemma in clinical practice [9, 10].

Plant extracts, honey, propolis, as well as larvae are traditional treatments for wound healing. Traditional remedies are popular throughout Latin America, Africa, as well as Asia. Leech therapy can debride wounds [11]. Honey, a natural sweetener, can be utilized as a wound dressing and helps re-epithelialize wounds [12]. *Aloe vera* and *Calendula officinalis *can improve wound closure and skin regeneration [13, 14].

Advanced contemporary wound healing procedures complement traditional therapy. In clinical settings, hyperbaric oxygen treatment saves amputation by improving intractable or complex wounds with oxygen flow [15, 16]. Negative-pressure therapy promotes healing by reducing edema, altering capillary perfusion, and speeding collagen synthesis and angiogenesis. Wound dressings minimize infection, enhance regeneration, and reduce wound base damage to maintain wound hydration. Hyaluronic acid (HA), chitosan (CS), silica gel, as well as collagen are extensively used in wound dressings [17]. Uncertainty about their mode of action, the potential for infection or allergic reaction, and variations in quality from batch to batch are all disadvantages of these therapies. There are still many issues to be resolved [18] in the areas of production techniques, rational utilization, safety, quality, policy, as well as effectiveness.

Metal-organic frameworks (MOF) are organic-inorganic hybrid materials consisting of metal ions/clusters and organic linkers. The assembly of these ingredients leads to multidimensional architectures from 0D to 3D with high potential to perform in a variety of applications, such as catalysis, gas capture/separation, photoluminescence, sensing, adsorption, and drug delivery [19-21]. These applications can be achieved by MOFs due to their numerous characteristics, including large surface area, large pore size/volume, crystallinity, porosity, modularity, functionality, and compatibility with other materials, forming an array of composite materials with augmented properties outperforming the pristine ingredients. 

Among the wide variety of applications, MOFs were employed in biological applications to first work as drug delivery carriers for some therapeutic agents, as presented by Horcajada and colleagues [22, 23]. The second is using MOFs themselves as active materials. However, many aspects should be considered regarding the toxicity and stability of the deployed MOFs in the biological environment. Several MOFs exhibit a large surface area (for loading cargo), facile surface modification (for biomedical purposes), and customizable pore diameters (for wrapping therapeutic agents) [24, 25]. MOFs are incredibly versatile since their structures may be tailored to suit a wide range of applications; also, their core metal components can accommodate a wide range of metal ions, such as cobalt (Co), zinc (Zn), as well as copper (Cu). Since the MOF degradation process involves the release of metal ions from the MOF centers, it may be advantageous to select a metal ion that aids in diabetic wound healing as the MOF carrier. Some review articles have been reported on wound healing; however, less has been reported on using MOFs for diabetic wound healing, according to our knowledge [26-28].

**Figure 1:**
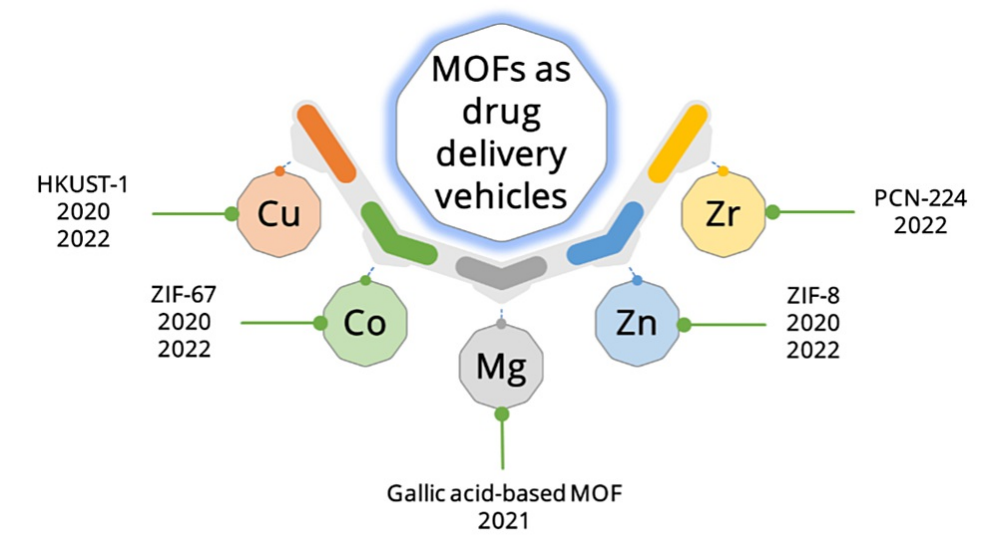
Timeline development of divalent- and tetravalent-based MOFs for diabetic wound healing. Cu, Copper; Zn, zinc; Co, cobalt; Mg, magnesium; Zr, zirconium; MOF, Metal-Organic Framework; ZIF, zeolitic imidazolate framework; PCN, porous coordination network

In this review, we summarize the use of divalent and tetravalent-based MOFs in diabetic wound healing (Figure 1). In most reports, MOFs are employed as drug delivery carriers for medical agents such as nitric oxide (NO). In NO-based therapy, MOFs can help control the release of NO to reach the wound area and improve healing in a short duration. In addition, metal ions released from the MOF framework can have a dual effect on the healing process.

## Review

Divalent-based MOFs

Cu-based MOFs

**Figure 2:**
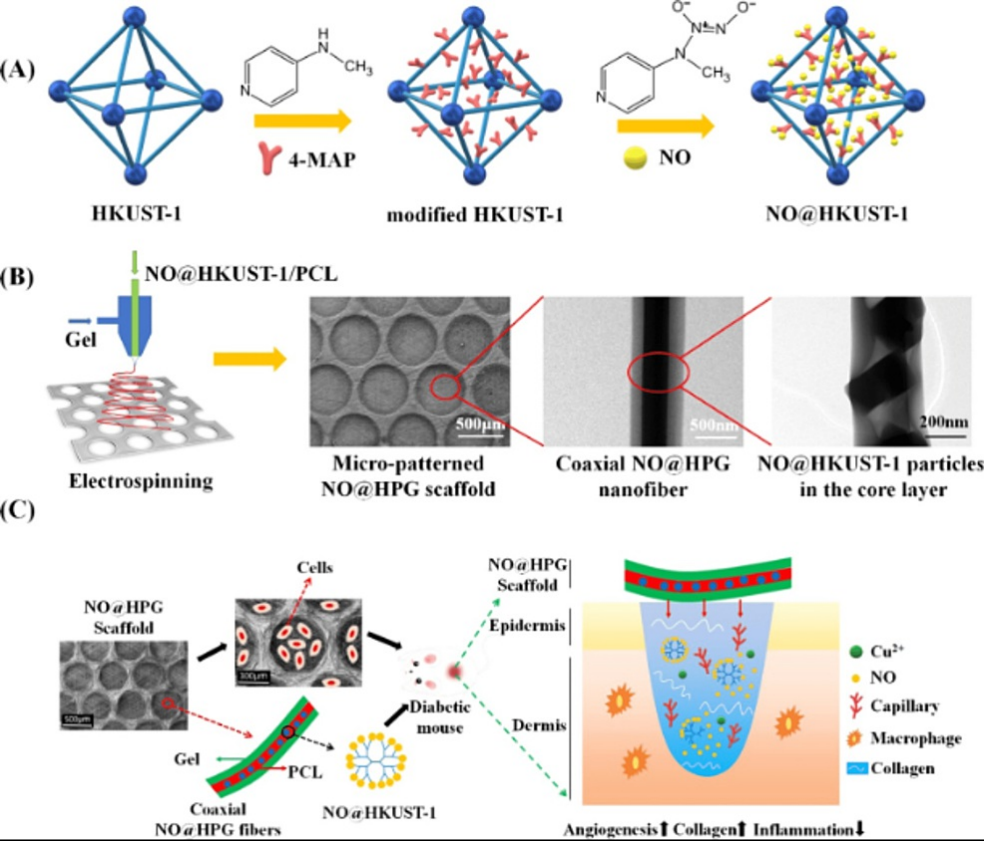
Synthesis of (A) NO@HKUST-1 and (B) NO@HKUST-1/PCL/Gel (NO@HPG) Scaffold. (C) Increased diabetic wound healing with a composite scaffold. Reproduced with permission from the American Chemical Society. PCL, hydrophobic polycaprolactone; NO, nitric oxide; HPG, Hyperbranched poly(glycidol); PCL, polycaprolactone; Cu^2+^, copper ion, 4-MAP, 4-(methylamino) pyridine

One of the most essential requirements for using MOFs in diabetic wound healing is the selection of MOF type based on its metal center and MOF properties to be compatible with the affected area. In this regard, Zhang et al. selected the famous copper-based MOF-HKUST-1 to be the carrier of NO for subsequent delivery under controllable release at the wound area. Typically, using coaxial electrospinning, a micropatterned electrospun scaffold was created, integrating NO@HKUST-1 as a controlled NO-releasing system (Figure 2) [29]. NO-loaded HKUST-1 revealed significant benefits over other NO donors, including great biocompatibility and biodegradability, storage stability, in addition to prolonged release half-life. Core-shell architecture endowed the scaffold with a continuous, low-level release of NO as well as copper ions. Typically, the outcomes showed that NO and copper ions could work together to boost collagen deposition, reduce inflammation, and speed up the healing process at wound sites by stimulating angiogenesis and promoting collagen synthesis. As a consequence of the controlled synthetic process, NO can be released at a steady rate of 1.74 nmol L-1 h-1 for over two weeks. Additionally, the released copper ions from biodegradable HKUST-1 are highly critical to provide a synergetic function with NO for boosting the growth of endothelial cells while greatly enhancing collagen deposition, angiogenesis, as well as anti-inflammatory characteristics in the wound bed, ultimately speeding up the healing of diabetic wounds. Such outcomes ensure that HKUST-1 is a highly auspicious framework for the controllable release of NO to quickly heal diabetic wounds.

**Figure 3:**
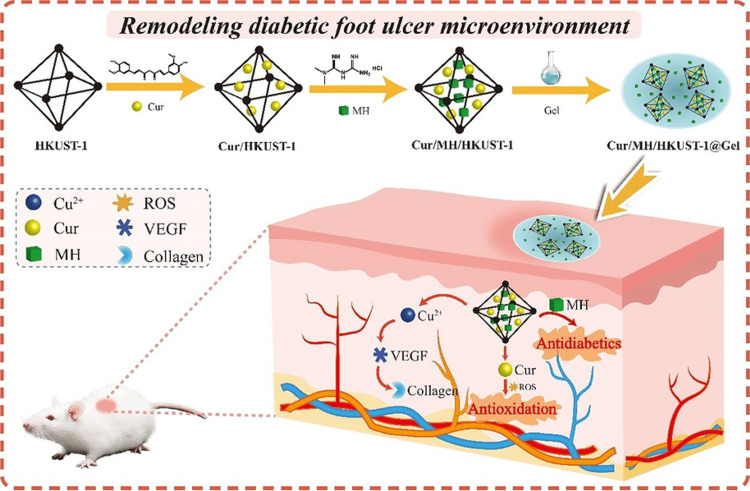
Fabricated Cur/MH/HKUST-1@Gel for treating murine diabetic wounds. Reproduced after seeking permission from Elsevier. Cu^2+^ copper ion; Cur, curcumin; MH, metformin hydrochloride; ROS, reactive oxygen species; VEGF, vascular endothelial growth factor.

Diabetic wounds are characterized by abnormal skin angiogenesis in addition to extended oxidative stress of the wound region, which prevents them from healing properly and poses a significant therapeutic challenge. Accordingly, we desperately need access to specialized wound dressing systems that promote healthy blood flow and reduce inflammation. Yang et al. presented a strategy for reshaping the microenvironment surrounding DFUs that uses a hybrid composite (Cur/MH/HKUST-1), in which HKUST-1 MOF was incorporated with metformin hydrochloride (MH) in addition to curcumin (Cur) (Figure 3) [30]. This hybrid composite underwent impregnation into thermosensitive hydrogel loaded with MH (called Cur/MH/HKUST-1@Gel). Particularly, dual drug load efficiency, bio-stability, as well as drug release patterns based on pH were identified as distinguishing features of Cur/MH/HKUST-1@Gel. This system was found to have therapeutic efficacy in human epidermal keratinocytes (HaCat)** **and human skin fibroblasts (HSF) cells, where it promoted migration and proliferation of granulation tissue cells while also inhibiting the effects of oxidative stress. Diabetic mouse wound healing is drastically sped up by Cur/MH/HKUST-1@Gel because it maintains continuous releasing of MH, Cur, as well as copper ion (Cu^2+^) and provides a humid microenvironment. Newborn blood vessels, increased cell proliferation, and abundant collagen fiber deposition were observed when Cur/MH/HKUST-1@Gel was employed to treat wounds, making it a highly promising appropriate system for DFU healing. This work offers the great potential of HKUST-1 as a reliable platform to be exploited for developing many suitable systems amenable to wound healing in general and to diabetes in particular.

**Figure 4:**
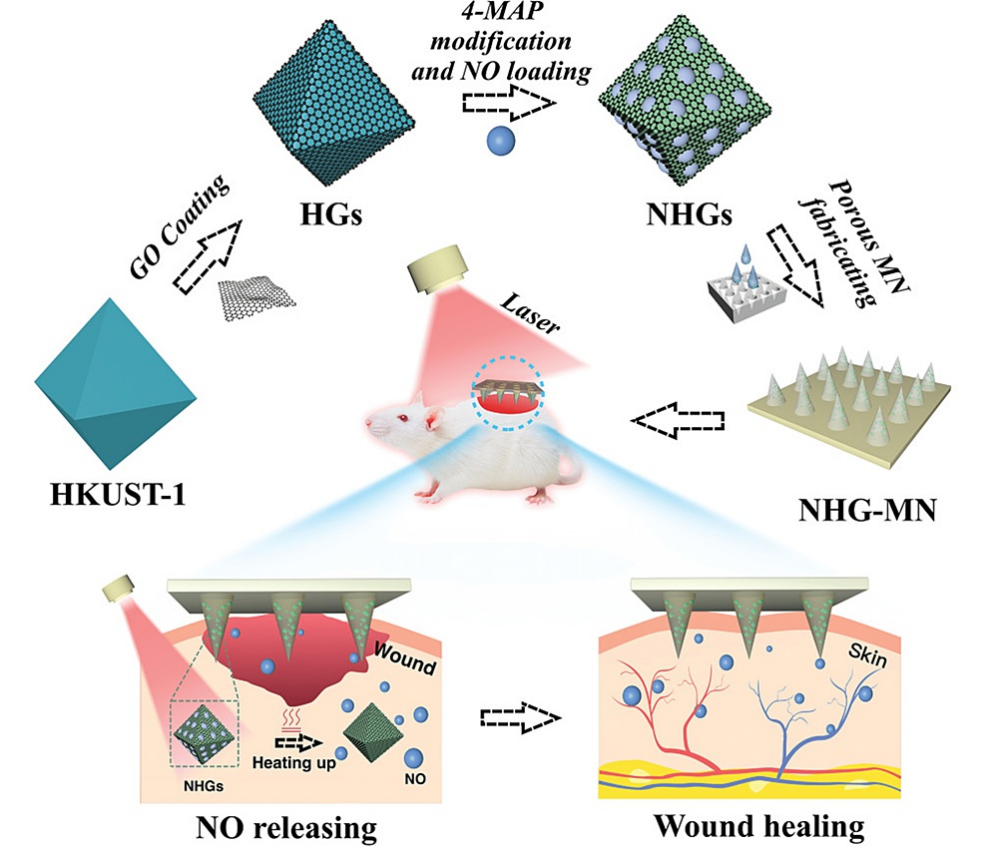
Applying porous MOF MN array in wound healing. Copyright © 2021 The Authors, Advanced Science, published by Wiley‐VCH GmbH MOF, Metal-Organic Framework; MN, microneedle; HG, hydrogel; NHG, nanohydrogen; GO, graphene oxide; 4-MAP, 4-(methylamino) pyridine

A new method of transdermal medicine administration, microneedles (MNs) are characterized by a dense array of needle-like projections on their surface that are only microns in size [31]. In order to assist the loading of active substances, porous MN based on pore-forming materials includes many pores/gaps in needle tips and base [32]. In particular, a number of distinct MOFs, including HKUST-1, were able to preserve NO adsorption and separation capacity [29, 33]. As a rule, such MOFs would break under the stress of a regulated NO discharge. The integration of MOF/MN patch for controlled release of gas molecules suggests a unique sophisticated release system for NO to treat refractory wounds [34]. To aid in the healing of diabetic wounds, Yao and coworkers proposed a new MOF/MN patch with distinct porosity that enables photothermal-responsive NO release (Figure 4) [35]. NO molecules underwent facile controlled release by the incorporation of near-infrared ray (NIR) photothermal response into NO@HKUST-1@GO microparticles (NHGs). Such integrated MN exhibited structural porosity and a higher surface area, in addition to appropriate mechanical strength with the great potential to endow the wound site with NO molecules in a precise and deep delivery pattern upon encapsulating such NHGs in a porous poly(ethylene glycol) diacrylate microneedle​​​​​​​ (PEGDA-MN). NHG-MN has the potential to accelerate tissue regeneration, vascularization, as well as collagen deposition by administering it to a type I diabetic rat model at the wound area, highlighting its potential in wound healing in addition to further therapeutic settings. In the above reports, HKUST-1 has proven much utility to be an excellent MOF candidate for subsequent integration with other materials to produce a unique platform as a vehicle for the controllable release of medicine gases to enhance/inhibit all attributes linked to the wound healing process. This in turn facilitates wound healing, especially in diabetes, which requires further exploration of other copper-based MOFs to inspect their potential behavior in diabetic wound healing.

Co-based MOFs

**Figure 5:**
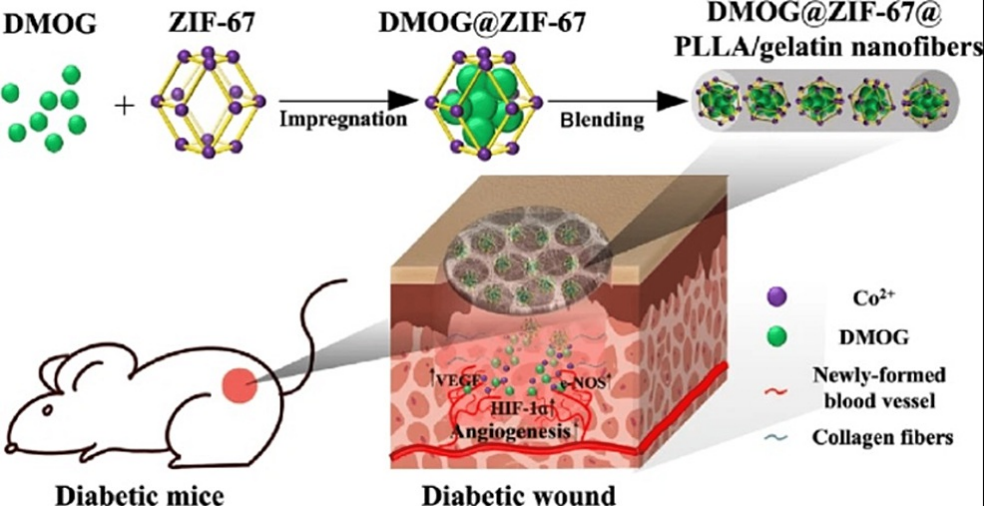
Using DMOG@ZIF-67@PLLA/gelatin nanofibers for diabetic wound healing. Reproduced with permission from Springer Nature. DMOG, dimethyloxalylglycine; ZIF-67, zeolitic imidazolate framework-67; PLLA, poly (L-lactic acid); Co^2+^, Cobalt ion

One of the most significant factors that contribute to a diabetic chronic wound's inability to heal on its own is a lack of angiogenesis in the wound.Li et al. developed a cobalt-based MOF known as zeolitic imidazolate framework-67, or ZIF-67, working as a carrier to load a small molecule medication that promotes angiogenesis called dimethyloxalylglycine (DMOG) (Figure 5) [36]. In order to accomplish angiogenic therapy in the long run, particularly on wound beds of diabetes, a designed system exhibiting dual cooperative controllable delivery was accomplished. This system was created using micro-patterned poly (L-lactic acid) (PLLA)/gelatin nanofibrous scaffolds that incorporate drug-loaded ZIF-67 nanoparticles. This system is aimed at achieving angiogenic therapy in the long run. The findings demonstrated that DMOG underwent loading onto ZIF-67 nanoparticles exhibiting a higher ratio of loading as of 359.12 mg/g. In addition, the results demonstrated that drug-loaded ZIF-67 nanoparticles were successfully encapsulated within a circularly patterned scaffold. Remarkably, both DMOG and the Co ions were able to continue to release from the scaffold for a period of time over half a month. According to in vitro studies, human umbilical vein endothelial cells' ability to proliferate, migrate, and form tubes was synergistically promoted when micropatterned nanofibrous scaffolds release Co ions and DMOG. This was demonstrated by the fact that these two factors had the ability to be released from the scaffolds (human umbilical vein endothelial cells​​​​​​​, HUVECs). This was accomplished through hypoxia response induction and upregulation of angiogenesis-related gene expressions, including hypoxia-inducible factor 1​​​​​​​ (HIF-1), vascular endothelial growth factor​​​​​​​ (VEGF), as well as endothelial nitric oxide synthase​​​​​​​ (e-NOS). In addition, the data obtained in vivo revealed that composite scaffolds were able to considerably improve collagen deposition and angiogenesis and remove inflammation in diabetic wounds. Such findings suggest that cobalt-based MOF functions as a dual cooperative controlled release platform, offering a novel method for boosting diabetic wound healing and enhancing angiogenesis.

**Figure 6:**
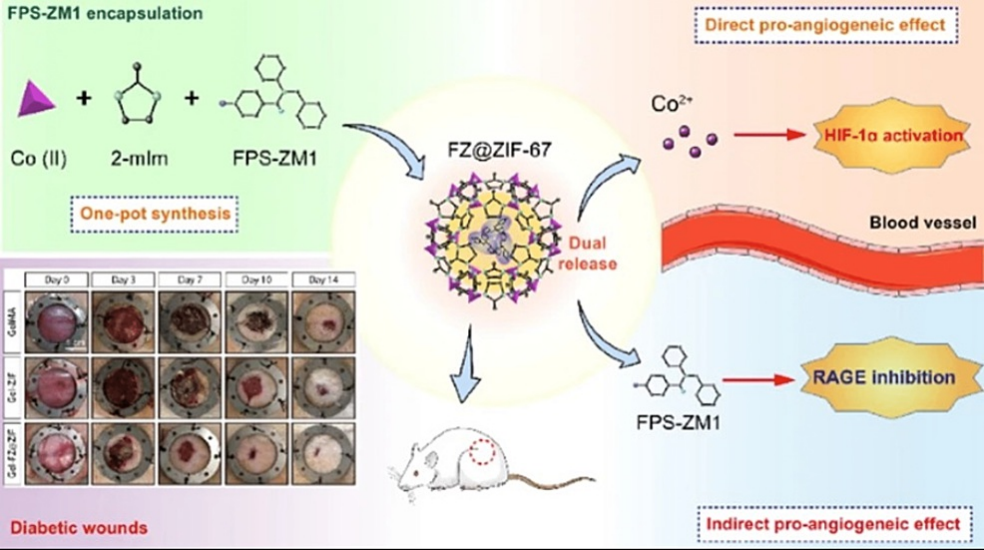
Using FZ@ZIF-67 NPs for diabetic wound healing. Reproduced with permission from Springer Nature. FPZ-ZM1, 4-chloro-N-cyclohexyl-N-(phenylmethyl)-benzamide; ZIF-67, zeolitic imidazolate framework-67; Co (II), Cobalt(II) oxide; 2-mlm, 2-methylimidazole; FZ@ZIF-67, FPS-ZM1 encapsulated ZIF-67; Co^2+^, Cobalt ion; RAGE, receptor for advanced glycation endproducts; HIF-1α, hypoxia-inducible factor 1-alpha

Diabetes-related wounds provide a great deal of therapeutic potential for inhibitors of signaling pathways that are pertinent to the pathogenesis of diabetes. MOFs are becoming increasingly attractive as drug delivery vehicles because of their high loading capacity and their ability to release the metal ions that are inherent to the framework for acting as bioactive agents. As a result of this, ZIF-67 based on cobalt metal centers was employed to load an inhibitor of receptor for advanced glycation end-products​​​​​​​ (RAGE)known as 4-chloro-N-cyclohexyl-N-(phenylmethyl)-benzamide (FPS-ZM1) for producing nanoparticles (NPs) of FPS-ZM1 encapsulated ZIF-67 (FZ@ZIF-67) (Figure 6) [37]. Because of this, these NPs were able to disperse Co ions as well as FPS-ZM1 in a sustained fashion for a period of more than 14 days. Through the use of an in vitro model, NPs improved angiogenesis through Co ion delivery, delivered FPS-ZM1 for promoting M2 macrophage polarization, as well as reduced the impairment of angiogenesis caused by a high-glucose (HG) environment and/or inflammation by inhibiting RAGE. All of these effects were brought about by the NPs. When injected into diabetic wounds in rats, FZ@ZIF-67 NPs dramatically augmented collagen deposition, neovascularization, re-epithelialization, as well as reduced inflammation, as shown in another study. In order to improve vascularization impairment that occurs during diabetic wound healing, it is necessary to target diabetes-related pathological signaling pathways. This was discovered in addition to the discovery of a bioactive chemical that is synergistic, efficient, and cost-effective in its proangiogenic properties.

Mg-based MOFs

**Figure 7:**
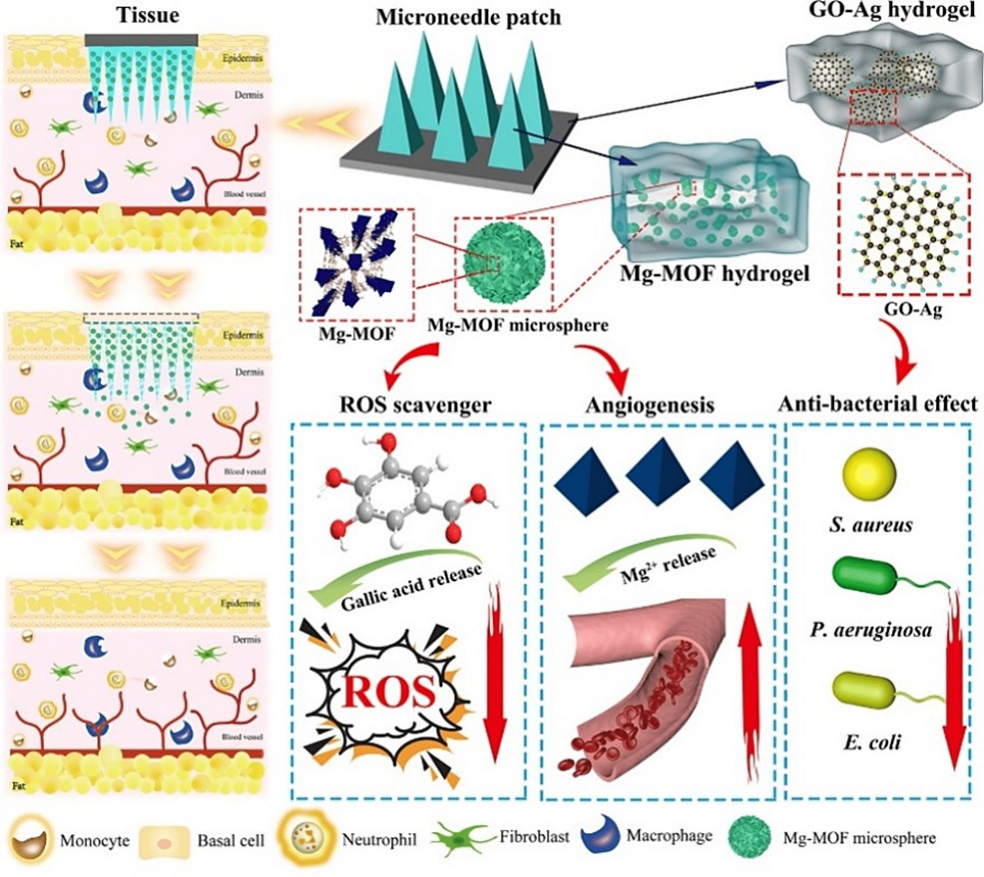
MN-MOF-GO-Ag for accelerated diabetic wound healing. Reproduced with permission from American Chemical Society. GO-Ag, graphene oxide-silver nanocomposites; Mg-MOF, Magnesium-based Metal-Organic Framework; ROS, reactive oxygen species; Mg^2+^, Magnesium ion

Healing wounds in diabetic patients is one of the most difficult problems to solve in biomedicine. The efficacy of traditional single-drug therapies is inadequate, and the effectiveness of drug administration is limited by the penetration depth. Yin and coworkers prepared a microneedle patch based on Mg-based MOF (MN-MOF-GO-Ag), featuring the capability of realizing transdermal distribution in addition to combination therapy for the treatment of wound healing in diabetes (Figure 7) [38]. Typical multifunctional Mg-MOFs, as well as poly(γ-glutamic acid) (γ-PGA) hydrogel, underwent mixing before being inserted into the MN-MOF-GO-Ag tips. This causes a gradual release of Mg^2+^ as well as gallic acid in the dermis's deep layer. Cell migration and endothelial tubulogenesis can be stimulated by the freed Mg^2+^, and antioxidation can be boosted by the presence of gallic acid, which is a scavenger for reactive oxygen species. Additionally, MN-MOF-GO-Ag exhibited a backing layer comprising γ-PGA hydrogel in addition to graphene oxide-silver nanocomposites (GO-Ag). Such a system provides outstanding antibacterial properties towards wound healing acceleration. For a diabetic mouse model, full-thickness cutaneous wounds were employed for illustrating MN-MOF-GO-Ag therapeutic effects on the process of wound healing. Treating mice using MN-MOF-GO-Ag causes an excellent improvement in the rate at which wounds heal.

Zn-based MOFs

**Figure 8:**
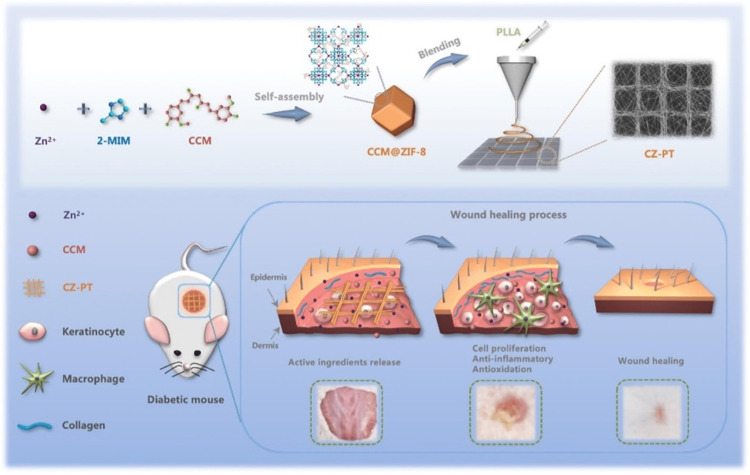
Fabricated porous patterned PLLA composite electrospun scaffolds incorporated with CCM@ZIF-8 MOFs for promoted diabetic wound healing. Reproduced with permission from Elsevier. Zn^2+^, Zinc ion; 2-MIM, 2-methylimidazole; CCM, curcumin; ZIF, zeolitic imidazolate framework; CZ-PT, composite scaffolds; PLLA, poly (L-lactic acid).

The main factors that contribute to diabetic patients' decreased wound healing include excessive persistent inflammation as well as oxidative stress. One of the most important goals of wound healing in diabetes is bringing down the levels of excess reactive oxygen species (ROS) in addition to wound inflammatory response during the acute inflammatory phase. Wang et al. used eutectic method to prepare Zn-based MOF using curcumin (CCM) as the ligand (Figure 8) [39]. CCM@ZIF-8 MOFs were then loaded into scaffolds of hierarchical micro/nanofibrous PLLA to create a regulated double release system comprising curcumin as well as Zn^2+^ for wound repair in diabetes. According to the findings, the synthetic MOFs were able to substantially increase curcumin bioavailability, and fabricated hierarchical micro/nanofibrous scaffolds were able to boost skin tissue cell adhesion and growth and deliver curcumin as well as Zn^2+ ^on-demand at the initial inflammation stage. Further in vivo investigations confirmed that scaffolds augmented the wound healing process in mice with diabetes by suppressing inflammatory response, increasing collagen deposition, fostering angiogenesis, in addition to re-epithelializing the lesion. ROS production, as well as inflammatory response in acute inflammation phase of wound healing, underwent great reduction by scaffolds. The underlying mechanism is closely related to this finding. Based on findings, hierarchical micro/nanofibrous scaffolds consisting of curcumin as well as Zn^2+^ eutectic MOFs may be an effective method for treating chronic wounds in diabetic mice.

**Figure 9:**
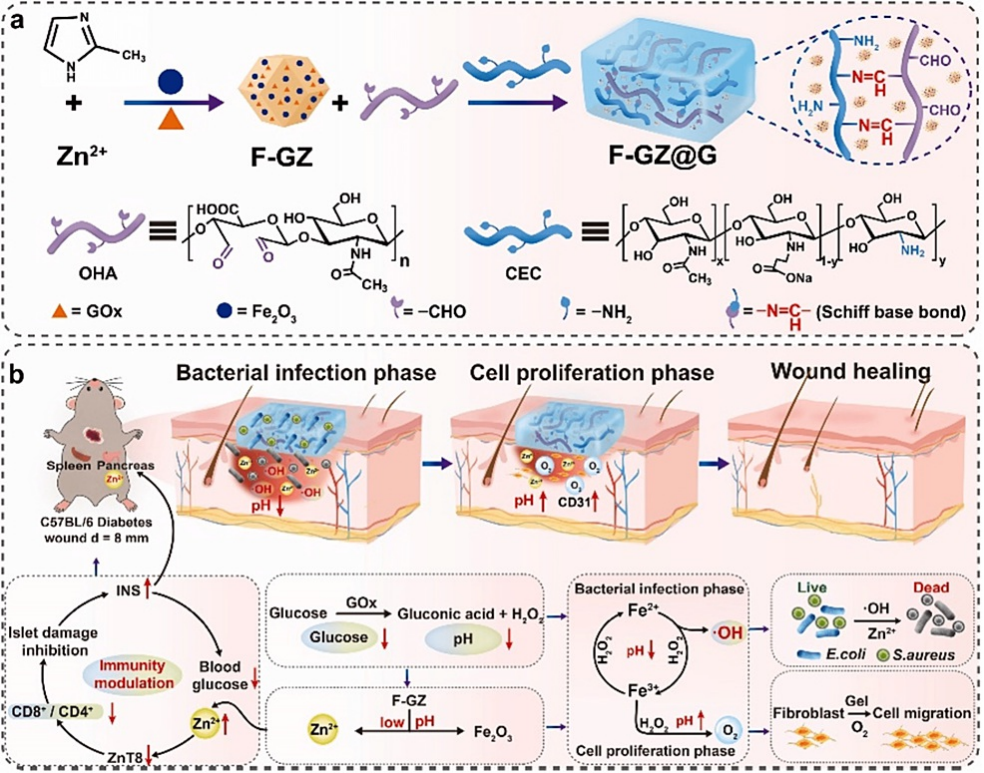
F-GZ@G synthesis and application. Preparation of (a) F-GZ@G and its (b) underlying mechanisms for diabetic wound repair as well as immunomodulation. Reproduced with permission from Elsevier. Zn^2+^, zinc ion; Fe2O3, Iron oxide; Gox, Glucose oxidase; ZIF-8, Zeolitic imidazolate framework-8; F-GZ, Fe2O3-GOx@ZIF-8; OHA, oxidized hyaluronic acid; CEC, N-carboxyethyl chitosan; -NH2, Primary amide group; H2N, Amino group; INS, Insulin; pH, potential of hydrogen (acidity or basicity of an aqueous solution); CD31, cluster of differentiation 31 (Platelet endothelial cell adhesion molecule); CD8 and CD4, protein markers that are commonly used as cell surface markers for T cells; H2O2, Hydrogen peroxide; Fe2+, Ferrous ions; Fe3+, Ferric cation; O2, oxide of hydrogen (Oxygen); E. coli, *Escherichia coli*; S. aureus, *Staphylococcus aureus*; ZnT8, zinc transporter 8; C57BL/6, a common inbred strain of laboratory mouse.

An essential problem still exists in the field of diabetic immunity-modulated wound therapy responding to diverse microenvironments that can be found at various phases. For their cascade enzyme catalytic activities, Deng et al. used Zn-MOF nanoparticles (F-GZ) co-incorporated with glucose oxidase (GOx) as well as quasi-amorphous ferric oxide (Fe_2_O_3_), in which increased blood glucose in wound undergoes consumption mostly through GOx catalysis and efficacious anti-bacteria undergoes accomplishment via degraded, delivered Zn^2+^ concurrently with catalytically generated hydroxide (OH) radicals in a low pH infection microenvironment (Figure 9) [40]. Catalyzing hydrogen peroxide (H_2_O_2_) to generate O_2_ in an environment with a relatively increased pH can simultaneously alleviate hypoxia and scavenge reactive oxygen species during the wound healing process. The copolymerization reaction then produces an injectable, self-healing, hemostasis-capable hydrogel with consistent F-GZ loading. The hydrogel has the same properties as F-GZ; however, it mitigates the toxic properties, which speeds up the healing process for diabetic wounds. Most notably, it was discovered that this hydrogel could modulate diabetes immunity, which may be mediated by the released Zn^2+^. This adds to recovered roles of pancreatic islets, which include enhanced glucose tolerance as well as greater insulin secretion towards better therapies for wounds in diabetes. The current research introduces an innovative technique towards simultaneously managing diabetic wounds while providing a possible mechanism towards diabetes immunity to be modulated.

Tetravalent-based MOFs

Zr-based MOFs

**Figure 10:**
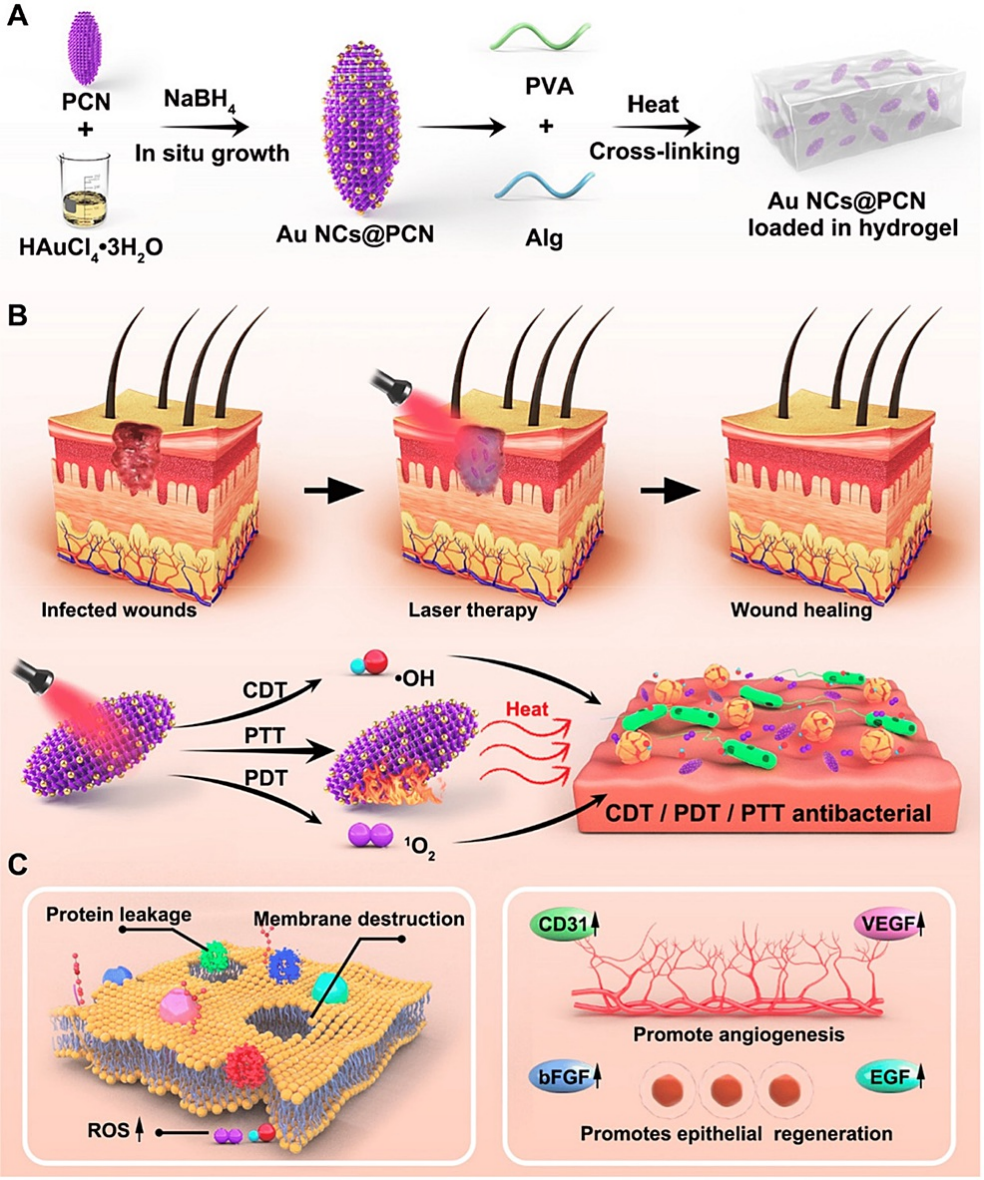
(A) Au NCs@PCN synthesis. (B) Using CDT, PDT, and PTT at high temperatures under NIR radiation, multidrug-resistant bacteria can be killed. (C) Bactericidal by causing membrane disruption and protein leaking in bacteria as well as enhancing angiogenesis and epithelial cell healing by increasing production of associated factors. Reproduced with permission from the American Chemical Society. Au NCs@PCN, gold nanoclusters modified zirconium-based porphyrin MOFs; HauCl4, chloroauric acid; ROS, reactive oxygen species; NABH4, Sodium borohydride; PVA, Polyvinyl alcohol; ALg, Alginate; CDT, chemodynamic therapy; PDT, photodynamic therapy; PTT, photothermal therapy; NIR, Near-Infrared radiation; bFGF, basic fibroblast growth factor; EGF, Epidermal growth factor; VEGF, vascular endothelial growth factor,

Diabetic wound healing has been significantly hampered by bacterial infection. It would be ideal if there was a multipurpose nanoplatform that could be utilized as a nanozyme to treat diabetic wounds infected with bacteria. Accordingly, an in-situ growth approach was used to create gold nanoclusters modified zirconium-based porphyrin MOFs (termed as Au NCs@PCN) (Figure 10) [41]. Scanning electron microscopy​​​​​​​ (SEM), transmission electron microscopy​​​​​​​ (TEM), as well as energy dispersive spectroscopy​​​​​​​(EDS)mapping revealed that the surface of ellipsoid-shaped particles with a size of about 190 nm was uniformly sprinkled with gold nanoclusters measuring between 5 and 8 nm in size. Most noteworthy, Au NCs@PCN performs exceptionally well in stimulating ROS production as well as photothermal effects. It has been shown that near-infrared (NIR) laser irradiation may raise the temperature of Au NCs@PCN to 56 °C, where they generate ROS and kill bacteria. The antibacterial effects of Au NCs@PCN were demonstrated by their ability to suppress the growth of methicillin-resistant *Staphylococcus aureus *(MRSA) and ampicillin-resistant (AMPr) *Escherichia coli *​​​​​​​(AmprE. coli)** **through damaging membrane structure as well as generating protein leakage at rates of 95.3% and 90.6%, respectively. Experiments on animals demonstrated that within three weeks, wound coverage decreased to 2.7% in diabetic rats treated with Au NCs@PCN. The immunoblot analysis demonstrated a dramatic increase in the expression of factors that promote the proliferation of both angiogenic and epithelial cells. Such findings demonstrate the potential utility of Au NCs@PCN for accelerating the healing of diabetic-infected wounds.

**Figure 11:**
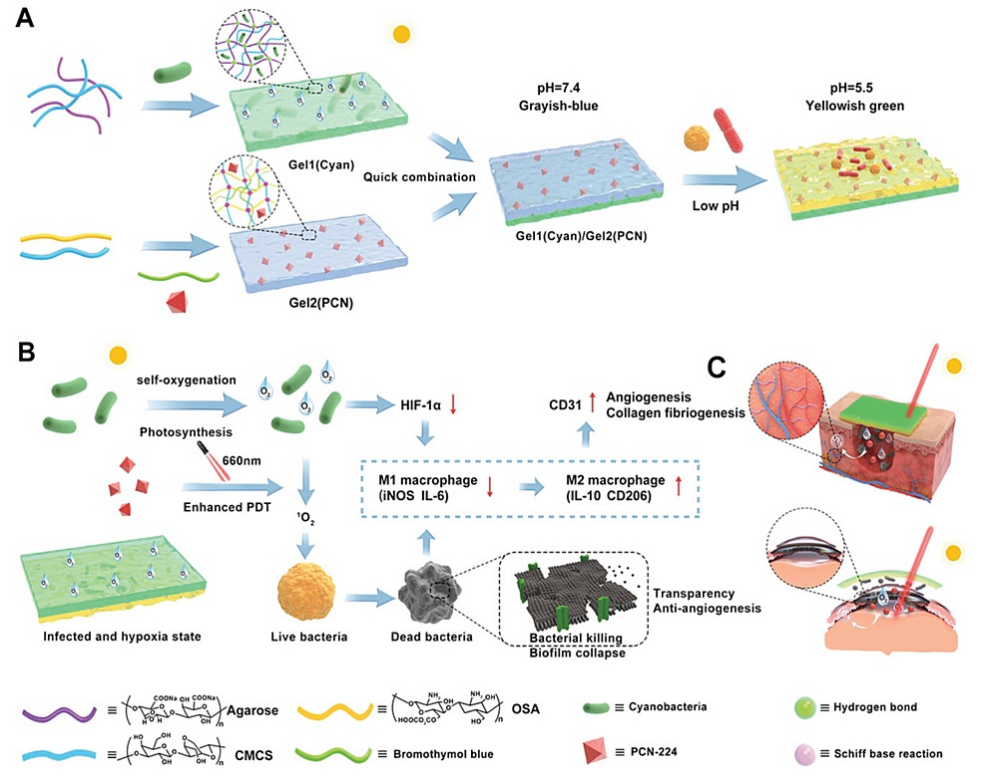
Preparing multifunctional double-layered hydrogel for the treatment of diabetic wounds as well as refractory keratitis. (A) Synthesis. (B) Multifunctional characteristics. (C) Implementation of double-layered hydrogel in healing diabetic wounds as well as refractory keratitis. Reproduced with permission from John Wiley and Sons. HIF-1-alpha, Hypoxia-inducible factor 1-alpha; IL 6, IL 10, Interleukin 6 & 10; iNOS, inducible nitric oxide synthase; OSA, octenyl succinic anhydride; CMCS, carboxymethyl chitosan; PCN, porous coordination network; PDT, photodynamic therapy

The danger of amputation, blindness, and death remains for patients with refractory keratitis as well as DFUs brought on by hypoxia, bacterial infections, in addition to chronic inflammation. Recently, Zhu and colleagues presented a double-layered hydrogel that generates oxygen, allowing us to determine where infections are and then use that information to tailor treatments like antimicrobial photodynamic therapy (PDT) as well as inflammation reduction to speed healing of diabetic wounds (Figure 11) [42]. Incorporating a photodynamic PCN-224 MOF as well as a pH indicator (bromothymol blue) into the inner layer of hydrogel (including oxidized sodium alginate/carboxymethyl chitosan [CMCS] through the use of a Schiff base) has proven to be effective. In order to combat tissue hypoxia and improve the efficacy of antimicrobial PDT, photosynthetic cyanobacteria were loaded into the outer hydrogel (made from agarose plus CMCS). Constant oxygen supply under natural light has its own set of benefits, including hastening cell migration, reducing inflammation, encouraging new capillaries to develop in the skin, and speeding up the healing of wounds. As a result, self-oxygenated double-layered hydrogel has substantial advantages to synergistically treat refractory anaerobe wounds, including the ability to monitor infection levels in real-time and promote tissue healing.

## Conclusions

In this review, we presented several MOF examples as drug delivery vehicles to accommodate medical agents such as nitric oxide (NO) to control their release at the wound area to accelerate the healing process. The review of the literature reveals that divalent- (Cu, Zn, Co, and Mg) and tetravalent (Zr)-based MOFs have been used as composite materials for diabetic wound healing in particular. Several examples of metal-organic materials can perform well in the healing process, such as using metal-organic cages or metallocavitands. Considering the toxicity and instability of MOFs under the process, selecting more non-toxic and biologically stable MOFs is highly required to allow for a safe process. In this regard, researchers have the ability to screen most porous metal-organic systems to figure out how they could be beneficial for the healing process. MOFs have several limitations or challenges that need to be addressed before they can be used in diabetic wound healing. These limitations include stability, potential toxicity, scalability of production, and translation to clinical applications. In the end, we hope this work to hint other colleagues for considering this area of research aiming at enriching our knowledge about one of the most urgent clinical issues that require much effort and innovation from multidisciplinary fields to be solved.
